# Investigating the Physicochemical and Antioxidant Properties of Goat Milk Enriched With Rice Extract Fermented by Exopolysaccharide-Producing Lactic Bacteria for Functional Yogurt Production

**DOI:** 10.1155/ijfo/8008452

**Published:** 2025-06-05

**Authors:** Mohammad Hossein Maleki, Milad Daneshniya, Farzaneh Abdolmaleki

**Affiliations:** ^1^Department of Food Science and Technology, S&R.C., Islamic Azad University, Tehran, Iran; ^2^Department of Food Science and Technology, Qa.C., Islamic Azad University, Qazvin, Iran

**Keywords:** antioxidant activity, fermented milk, goat milk, probiotics, rice extract

## Abstract

In recent years, the development of functional dairy products incorporating plant-based ingredients has gained considerable attention due to growing consumer interest in health-promoting foods. In this regard, the present study is aimed at developing probiotic fermented goat's milk using *Lactiplantibacillus plantarum* (ATCC 1058) and *Lacticaseibacillus casei* (ATCC 39392) strains. A key focus was on assessing the impact of rice extract (RE) incorporation on the viability and metabolic activity of these probiotic strains. To achieve this, goat's milk samples were inoculated with a probiotic mixture containing both strains at a concentration of 109 log colony-forming units (CFUs)/mL. Various concentrations of RE (0%, 2%, 4%, and 6%) were introduced to the milk samples. The ensuing fermented milk samples were then refrigerated for 28 days, during which assessments were conducted at 7-day intervals. Parameters under investigation included probiotic viability, acidity, pH, acetic acid content, L-lactic acid content, protein content, reducing sugar levels, Brix degree, viscosity, antioxidant potency, color attributes (brightness, yellowness, and redness), and sensory attributes. The study revealed significant changes in the physicochemical traits of the fermented milk during storage. Notably, acidity, L-lactic acid, acetic acid, and viscosity increased, while pH, reducing sugars, Brix, antioxidant strength, and probiotic levels decreased (*p* ≤ 0.05). The addition of RE and its increasing concentration intensified these shifts. Importantly, this effect corresponded with improved antioxidant activity and probiotic viability (*p* ≤ 0.05). The sample with 6% RE displayed the highest antioxidant potency (55.46%) and probiotic count (9.21 log CFU/mL) at the study's end, followed by the 4% RE sample. Furthermore, sensory assessment revealed that the 6% RE sample had the lowest acceptability score, whereas the other samples were well-received. Ultimately, with the provided outcomes, the optimal treatment for producing probiotic fermented milk with desirable functional attributes was determined to be a 4% concentration level of RE.

## 1. Introduction

In the past, the primary focus of food science experts was centred around achieving dietary equilibrium. One method employed to achieve this equilibrium was ensuring an adequate intake of nutrients while avoiding substances that could disrupt the dietary balance, such as excessive consumption of fats, cholesterol, and sodium. However, this approach has presented challenges. Currently, the discourse is shifting towards determining the optimal intake of nutrients to extend the average lifespan and identifying beneficial constituents within the composition of food. When incorporated into the diet, these constituents can potentially mitigate diseases and enhance overall health. One strategy to achieve this goal is the utilization of functional foods [1]. Functional foods first emerged in Japan during the early 1980s, and their prominence has grown steadily since then. Recent research has been instrumental in unveiling the correlation between diet and the prevention of severe and chronic illnesses. Additionally, factors such as the aging population in developed nations and the heightened concern for preserving the well-being of elderly individuals, who are more susceptible to ailments like cancer, osteoporosis, diabetes, heart conditions, and stroke, have further fuelled interest in functional foods [2]. Given the aforementioned considerations and the escalating consumer demand for convenient food options, the market is flooded with a diverse array of functional food products. These encompass a wide spectrum, ranging from sugary beverages like energy drinks, breakfast cereals, and children's food to cooked meals, confectioneries, dairy products, notably yogurt and various fermented dairy items, high-fat spreads, meat-based products, and animal feed [3].

Goat milk is gradually gaining popularity on a global scale. This particular milk variety boasts a multitude of advantages and is replete with essential nutrients. Remarkably, even individuals with milk allergies can partake in its consumption, as it is easily digestible and poses no significant challenges. Notably sought after for its flavor and health-promoting properties, goat milk has secured a prominent position among the most widely ingested milk types across the globe. Its economic viability is underscored by its comparative affordability, attributed to its exemption from the homogenization process. This milk variety stands as an exemplary source of nourishment, teeming with vital nutrients, vitamins, and minerals. Among its constituents are carbohydrates, proteins, and sodium. Additionally, goat's milk is fortified with essential minerals such as calcium, magnesium, potassium, copper, and zinc [4].

In addition to these benefits, its fermentation can enhance its nutritional value. Fermenting milk represents a relatively straightforward, cost-effective, and safe technique for its preservation. The practice of fermenting milk dates back to ancient times, with Central Asia regarded as its main place of origin. During the fermentation process, lactic bacteria convert lactose into lactic acid, resulting in a decrease in milk's pH (within the range of 4–4.6). This pH adjustment effectively hampers the growth and survival of harmful pathogens. Moreover, the reduction in lactose content makes these products suitable for individuals with lactose intolerance [5].

Rice extract (RE), derived from the rice grain, is a potent natural ingredient known for its rich composition and diverse health benefits. This extract is abundant in essential vitamins such as Vitamin E, which provides antioxidant protection against oxidative stress, and B vitamins like thiamin (B1) for energy metabolism, niacin (B3) for skin and nervous system health, and B6 for brain and immune function. Additionally, RE contains significant antioxidants, including gamma-oryzanol, tocotrienols, and tocopherols, which help reduce inflammation, oxidative damage, and support cardiovascular health. Phytosterols present in RE are effective in lowering LDL cholesterol levels and promoting heart health. The balanced mix of unsaturated and saturated fatty acids in RE, including Omega-3 and linolenic acid, is vital for brain health and maintaining a healthy inflammatory response. Moreover, the dietary fiber content aids digestion and promotes regular bowel movements. These properties make RE a valuable addition to both dietary supplements and skincare products, offering moisturizing, anti-aging, and soothing benefits for sensitive skin [6].

Exopolysaccharide (EPS)-producing lactic acid bacteria (LAB) enhance the texture, viscosity, and stability of fermented dairy products. EPS also supports probiotic survival during storage by forming protective matrices. Therefore, using EPS-producing strains like *Lactiplantibacillus plantarum (Lpb. plantarum)* and *Lacticaseibacillus casei (Lc. casei)* aligns with the goal of improving the functional and physicochemical qualities of probiotic fermented milk [7]. Some previous studies possess some similar aspects to the current study. In a study where the subcritical water extraction method was optimized for rice milling byproducts, efficiently produced bioactive compounds-total phenolics, flavonoids, organic acids-for healthy cereal-based milk analogs, which, when fermented with *Lc. casei* or *Lpb. plantarum* and combined with inulin and Persian grape molasses, increased nutrient levels—vitamins B3, B6, and B9—and supported LAB viability (106–108 CFU/mL) during storage, with *Lc. casei* enhancing bioactive compound production and antioxidant activity more significantly, though *Lpb. plantarum* beverages were preferred overall [8]. In another study on fermented milk with rice powder (4%), *Lycium chinense* Mill, and red ginseng extract revealed that the addition of rice powder led to a pH decline to 4.6 after 12 h, enhanced LAB growth, increased viscosity, sugar content (lactose, galactose, glucose, fructose), and organic acids—oxalic, malic, lactic, acetic, and isobutyric—with total phenol and flavonoid levels rising over time, though sensory preference decreased with more additives, while all samples remained stable during 15 days of storage [9]. In the study conducted by Boêno et al. [10], fermented dairy beverages with red RE and whey were developed, showing high protein, zinc, manganese, and copper levels, safe pH for 28 days without preservatives, pathogen-free status, viable LAB, and optimal formulations containing 20%–34% red RE and 26%–54% whey due to favorable copper content. By utilizing rice milling by-products and Persian grape molasses, Ardali et al. [11] developed functional milk analogs via subcritical water extraction and fermentation with *Lc. casei* and *Lpb. plantarum*, exhibiting promising replacements for milk ingredients and sucrose, with favorable rheological properties, sustained LAB viability, initial increases in total phenolic compounds and antioxidant activity during fermentation followed by a decline during storage, and highest overall acceptability for *Lpb. plantarum* drinks in sensory evaluation after 28 days.

Nevertheless, current research is unique in the majority of its aspects, aiming at producing a novel probiotic yogurt. By integrating *Lpb. plantarum* and *Lc. casei* as probiotic strains, the research evaluates the effects of varying RE concentrations (0%, 2%, 4%, and 6%) on probiotic viability, physicochemical properties, antioxidant activity, and sensory attributes over a 28-day storage period. Unlike prior studies, this investigation provides a comprehensive analysis of fermentation dynamics, highlighting the influence of RE on lactic and acetic acid production, viscosity, and antioxidant potency, while optimizing sensory acceptability. The findings are aimed at establishing a scientifically validated formulation that balances health-promoting properties with consumer preferences, contributing to the development of sustainable, nutrient-dense, and palatable functional dairy products.

## 2. Materials and Methods

### 2.1. Preparation of Tested Strains

The bacterial strains *Lc. casei* (ATCC 39392) and *Lpb. plantarum* (ATCC 1058), originally isolated from milk products obtained from local dairy distributors across Qazvin (Ghazvin Province, Iran), were provided in 50 g packets of lyophilized powder (10^12^ CFU/sachet). These strains were authenticated by the Persian Type Culture Collection (PTCC), Iranian Research Organization for Science and Technology. They were used as microbial starters in the preparation of fermented milk [12].

### 2.2. Preparation Method of Strains

The *Lc. casei* and *Lpb. plantarum* were cultured in MRS broth (Merck, Germany) to prepare the inoculum and incubated for 24 h at 37°C. The viable cell count of the inoculum was determined using the standard plate count method on MRS Agar (Merck, Germany), with incubation for 48 h at 37°C [13].

### 2.3. Evaluation of the Probiotic Activity of Strains

The evaluation of probiotic activity involves assessing key characteristics to ensure efficacy and safety. Acid resistance requires treated counts not falling below 10^6^ CFU/mL. Microorganisms are cultured in pH-adjusted media (4.0 and 2.5) for 3–4 h and then plated on MRS agar to determine CFUs [14]. For bile salt resistance, the inhibition coefficient must be < 0.4%. OD readings are taken at 600–650 nm before and after an 8 h incubation with bile salts [14]. Gastric juice resistance requires treated counts > 10^6^ CFU/mL. Microorganisms are cultured in gastric juice-simulating media with pepsin and trypsin, sampled at intervals (0–24 h), and CFUs are quantified [15]. The lack of hemolytic activity is confirmed using sheep blood agar; clear halos indicate *β*-hemolysis, with *Staphylococcus aureus* (ATCC 25923) as a positive control [14]. L-arginine hydrolysis is assessed by culturing microorganisms in arginine-containing media. A reddish-orange hue with Nessler's reagent indicates a positive reaction, with *S. aureus* as the control [15]. Finally, the enumeration of probiotic microorganisms verifies CFU counts per gram using serial dilutions and MRS agar plating [14].

### 2.4. Production of Probiotic Goat Milk Yogurt

A composite blend of 0.5 g, containing *Lc. casei* (ATCC 39392) and *Lpb. plantarum* (ATCC 1058), with each strain contributing approximately 10^9^ CFU, was introduced into sterile cylindrical glass containers (borosilicate, 250 mL capacity, 5 cm diameter × 13 cm height). Each container held 200 mL of pasteurized goat milk (KAICO, Iran) enriched with rice extract (RE) (Behmalt, Iran) at varying concentrations of 2%, 4%, and 6%. A control sample with no RE was also prepared. The containers were loosely sealed with sterile, gas-permeable covers (parafilm with pinholes) to allow proper gas exchange during fermentation while minimizing contamination. Fermentation was carried out at 37°C in an incubator until the pH of the fermented milk reached 4.5. Following fermentation, the containers were transferred to a refrigerator maintained at 5 ± 1°C for subsequent analysis and storage. The assessment of the fermented milk samples occurred on the first, seventh, 14th, 21st, and 28th days of refrigerated storage [16].

The mentioned concentrations for RE were selected based on a pretest that considered the viscosity and sensory attributes of the produced yogurt, following an arithmetic sequence starting at 2% with a common difference of 2%, up to 12%. The pretest results indicated that RE concentrations exceeding 8% resulted in deviations in the viscosity and sensory properties of the yogurt from the commonly accepted standards for this product. To ensure the study remained within a reliable range, the maximum RE concentration was limited to 6%.

### 2.5. Tests Performed on Yogurt

#### 2.5.1. Viability of Probiotics

At first, the samples were diluted in sterile Ringer's solution (Merck, Germany), and viable *Lactobacillus acidophilus* probiotic species were evaluated by pour plate culture on MRS Agar (Merck, Germany) culture (the antibiotic used was vancomycin.). The plates were placed in closed containers in an anaerobic incubator. An anaerobic detector was used to control anaerobic conditions. After 72 h of incubation at 37°C, the colonies were counted and expressed as log CFUper milliliter [17].

#### 2.5.2. Measurement of Acidity and pH

The pH values were measured using an electronic pH meter (Iskra, Kranj, Slovenia). For this purpose, the pH meter device was adjusted with a buffer solution (Mina Tajhiz, Iran) at a temperature of 20°C before use. Then, the sample was poured into a 50-mL beaker, the electrode of the device was placed entirely inside the sample, and the pH was read and recorded. The amount of acidity was also measured using the standard solution of sodium hydroxide and phenolphthalein (Mina Tajhiz, Iran) as reagents. For this purpose, the amount of 10 mL of the sample was weighed in a beaker, and the same weight of distilled water was added. Then, 0.5 mL of phenolphthalein was added and titrated with 0.1 normal sodium hydroxide (Mina Tajhiz, Iran) until a pink color appeared, and the percentage of acidity was obtained from Equation (1) [18].

(1) $$\begin{array}{c} \text{Titratable acidity }\left(\%\right)=\frac{N\times 0.009\times 100}{M}, \end{array}$$

where *N* is the amount of sodium hydroxide utilized and *M* is the weight of the sample.

#### 2.5.3. Measurement of Acetic Acid and L-Lactic Acid

Acetic acid and L-lactic acid were measured in fermented milk samples using the HPLC method. For this purpose, an Aminex HPX-87H column with dimensions of 7 × 300 mm and dilute sulfuric acid of 0.005 mol/L was used as the mobile phase with a constant flow of 0.6 mL/min. Five mL of the sample was added to 35 mL of sulfuric acid and centrifuged for 10 min at 4°C at 5000 rpm. The supernatant was filtered with a 0.45 *μ*m polycarbonate membrane before chromatographic analysis. The organic acids were separated at 0.6 mL/min at 65°C [19].

#### 2.5.4. Measurement of Protein Content

To determine total nitrogen content, the macro Kjeldahl method was employed. Glass balls, 10 g potassium sulphate, 0.5 g mercury oxide, and 5 g of sample were weighed with milligram precision and combined with 20 mL sulfuric acid in a Kjeldahl flask. The mixture was heated gently until foaming ceased, then at higher heat until clear, followed by 1.5 h of boiling. After cooling, 150 mL of distilled water and boiling chips were added, mixed, and cooled further. Separately, 50 mL of boric acid and four indicator drops were prepared. The Kjeldahl flask contents were mixed with 80 mL caustic soda, attached to a cooling system, and distilled. The distillate was titrated with 0.1 N hydrochloric acid. Nitrogen content was calculated using Equation (2), and protein content was derived by multiplying nitrogen by 6.38 [20].

(2) $$\begin{array}{c} \text{Total nitrogen}=\frac{\left(V_{\text{as}}-V_{\text{ac}}\;\right)\times A_{N}\times 1.40}{W_{S}},\\ \text{Protein Content }\left(\%\right)=\text{Total Nitrogen }\left(\%\right)\times 6.25, \end{array}$$

where *V*_as_ is the volume of acid utilized for the sample, *V*_ac_ is the volume of acid utilized for the control, *A*_*N*_ is the normality of acid, and *W_S_* is the sample weight.

#### 2.5.5. Measurement of Reducing Sugars

To determine the concentration of reducing sugars in fermented milk samples, an initial step involved adding 2 mL of the sample into a 100-mL flask. This was followed by dilution with water to reach the desired volume, ensuring thorough mixing for uniformity. Subsequently, 10 mL of the diluted sample was transferred to an Erlenmeyer flask. To this flask, 25 mL of Luff Schoorl reagent was introduced. The mixture was subjected to boiling in a hot water bath for 10 min. Following the boiling step, 15 mL of 20% potassium iodide and 35 mL of H_2_SO_4_ were added. The sample underwent titration using 0.1 normal sodium thiosulfate until the appearance of a yellow color. Subsequently, amylose was added, and a further titration ensued until the blue color disappeared. In parallel, a blank sample containing 25 mL of Luff Schoorl reagent was prepared. The quantification of reducing sugars in the samples was achieved by utilizing Equation (3) as described by Hartati [21].

(3) $$\begin{array}{c} \text{Reducing sugars }\left(\%\right)=\frac{V_{\text{th}}\times N\times 100}{W_{s}}, \end{array}$$

where *V*_th_ is the amount of utilized sodium thiosulfate, *N* is the normality, and *W*_*s*_ is the sample weight.

#### 2.5.6. Measurement of Dissolved Solids Content or Brix Degree

The Brix degree of fermented milk samples was measured using a refractometer (Portable PLA-2, ATAGO, Japan).

#### 2.5.7. Evaluation of Viscosity

To assess the viscosity of fermented milk samples, a viscometer (Brookfield, United States) equipped with a spindle numbered 64 and set to operate at a shear speed of 25 rpm was employed. The viscosity measurements were conducted at a controlled temperature of 10°C to ensure stable conditions as outlined by Achanta et al. [22].

#### 2.5.8. Measurement of Antioxidant Activity

The assessment of antioxidant activity in fermented milk samples was conducted using the 2,2-diphenyl-1-picrylhydrazyl (DPPH) radical scavenging method. Initially, a solution of 0.1 mM DPPH (Merck, Germany) was prepared in methanol. Subsequently, 0.1 mL of the sample was blended with 2.9 mL of the DPPH solution within a laboratory tube. The mixture was then subjected to vortexing at a speed of 500x for 120 s, followed by a resting period of 30 min. The resulting mixture's absorbance was measured using a spectrophotometer at a wavelength of 517 nm. The level of antioxidant activity exhibited by the samples was quantified as a percentage, aligning with the method described by Kalhor and Abdolmaleki [23].

#### 2.5.9. Evaluation of Colorimetric Indices

Colorimetric indices were assessed using a calorimeter (Waters, United States). This evaluation involved employing standard black-and-white reference sheets. For the white sample, which had a height of 1.5 cm, the calibration was established against standard sheets with coordinates *Y* = 83.32, *X* = 81.26, and *Z* = 98.03. The color evaluation followed the CIE (Commission Internationale de l'Éclairage) system [24]. In this system, the following parameters were measured: *L*∗ (lightness) ranged from 0 (black) to 100 (white), *a*∗ (green to red) ranged from −120 to 120, and *b*∗ (blue to yellow) ranged from −120 to 120.

#### 2.5.10. Sensory Evaluation

A sensory assessment was conducted with the involvement of 15 trained panellists from the Karaj Agricultural Engineering and Technical Research Institute (Karaj, Iran). These panellists undertook the task of evaluating the production samples. The assessment encompassed various attributes, including aroma, taste, texture, color, and overall desirability. The sensory characteristics were appraised using a five-point hedonic test scale, where scores were 4 to a good sample, 3 to an average sample, 2 to a bad sample, and 1 to a very bad sample [25].

### 2.6. Data Analysis

The experimental setup employed a completely randomized design consisting of four treatments, each replicated three times and the results are presented as mean ± SD. The comparison of means was performed utilizing Duncan's multirange test at a significance level of 0.05. The analysis was conducted using statistical software (SPSS 22.0). For sensory attributes evaluation, the principal component analysis (PCA) model was used for data projection; PCA was performed using XLSTAT (ver. 2022), with standardization carried out using the *n* − 1 approach to ensure the equal contribution of all variables regardless of their original units. The PCA model was constructed based on the Pearson correlation matrix, with a maximum of five factors considered to explore the dimensional structure of the dataset. Significant components were identified based on eigenvalues greater than 1 (Kaiser's criterion), and variables with loadings exceeding ± 0.5 were considered to contribute meaningfully to the components. A significance level of *α* = 0.05 was used for interpreting the results, ensuring statistical validity.

## 3. Result and Discussion

### 3.1. Evaluation of Probiotic Activity of *Lc. casei* and *Lpb. plantarum* Strains

The evaluation results of the probiotic strains are summarized in Table 1, revealing no significant differences in the characteristics of *Lc. casei* and *Lpb. plantarum* strains (*p* ≥ 0.05). Further analysis highlights distinct physiological traits exhibited by each probiotic strain, underscoring *Lc. casei* and *Lpb. plantarum* bacteria as promising probiotic candidates. In another investigation, strains *L. casei* (MYSRD 108) and *Lpb. plantarum* (MYSRD 71), recognized as potential probiotics, demonstrated robust probiotic attributes. Aligning with the results of the current study, Divyashree et al. [26] investigated *Lc. casei* (MYSRD 108) and *Lpb. plantarum* (MYSRD 71) strains and identified them as potential probiotics, showcasing bold probiotic properties, including survival in acidic pH, bile tolerance, autoaggregation, favorable cell surface properties, and susceptibility to various clinically effective antibiotics.

### 3.2. Viability of Probiotics in Fermented Milk

**Figure 1:**
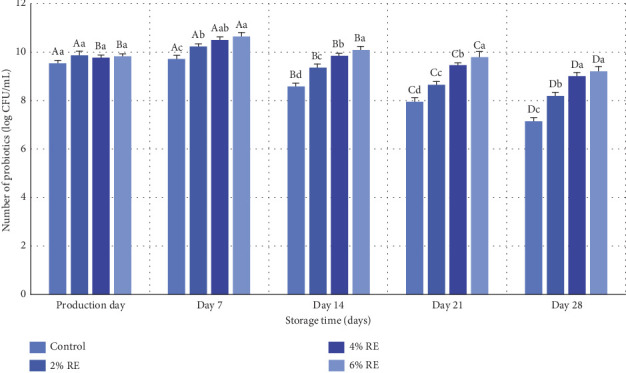
Comparison of the average number of probiotics (log CFU/mL) in fermented milk treatments during 28 days of storage time in the refrigerator. Different small letters denote significant differences among treatments at the same intervals, while different capital letters highlight significant differences within each treatment during storage.

The current study examined the viability of probiotics in fermented milk, revealing statistically significant effects of treatment, storage time, and their interaction on probiotic viability (*p* ≤ 0.05), as determined through variance analysis. A detailed summary of the results is presented in Figure 1. On the day of production, there were no statistically significant differences in probiotic counts among the various samples, indicating that the inclusion of RE had negligible effects on probiotic bacteria at this early stage. Probiotic counts ranged between 9.54 and 9.87 log CFU/mL on production day. However, during the storage period from production to Day 7, probiotic counts increased progressively. Subsequently, from Day 7 to Day 28 of refrigerated storage, a gradual decline in probiotic counts was observed across all samples (*p* ≤ 0.05), with the lowest counts recorded at the end of the storage period, ranging from 7.15–9.21 log CFU/mL.

Despite this decline, all treatments maintained probiotic counts above the minimum threshold of 6 log CFU/mL, meeting the criteria for probiotic products as defined by Massoud et al. [27]. The reduction in probiotic viability during storage is likely attributable to factors such as the accumulation of harmful metabolites, changes in water activity, pH shifts, oxidation-reduction potential (Eh), and the influence of antioxidant properties inherent to fermented milk, as well as variations in oxygen partial pressure [28].

Notably, the inclusion of RE improved probiotic viability, with higher concentrations of RE yielding more pronounced benefits. Samples with 6% RE showed the most tremendous increase in probiotic viability during storage. These findings align with Aiello et al. [29], who reported enhanced survival of LAB in probiotic products like kefir through the addition of plant-based natural extracts. This improvement was attributed to bioactive secondary metabolites present in the extracts [29]. Similarly, Abdollahzadeh et al. [28] demonstrated that date extract increased *Lactobacillus acidophilus* viability in fermented milk, while Ahmad et al. [30] observed that apple polyphenols significantly improved the survival of *L. acidophilus* in yogurt during storage. Ahmad et al. [30] emphasized that the prefermentation addition of apple polyphenols facilitated even probiotic dispersion, enhancing their survival compared to controls. Further supporting evidence comes from Zhao and Shah [31], who investigated the effects of black tea extract on *Bifidobacterium longum* in soy milk fermentation. They found that tea extract modified the bacterial cell membrane's fatty acid composition, particularly the saturated-to-unsaturated fatty acid ratio, and altered phosphatidylcholine and phosphatidylglycerol levels, ultimately enhancing probiotic viability [31].

In conclusion, the integration of natural extracts, such as RE and polyphenols, has consistently been shown to improve probiotic viability in fermented products. This enhancement is primarily attributed to bioactive secondary metabolites that influence cell membrane composition and facilitate probiotic dispersion. The findings of this study further underscore the potential of natural extracts as effective agents for improving the stability and viability of probiotics in functional food products during storage.

### 3.3. The pH Value of Fermented Milk

**Figure 2:**
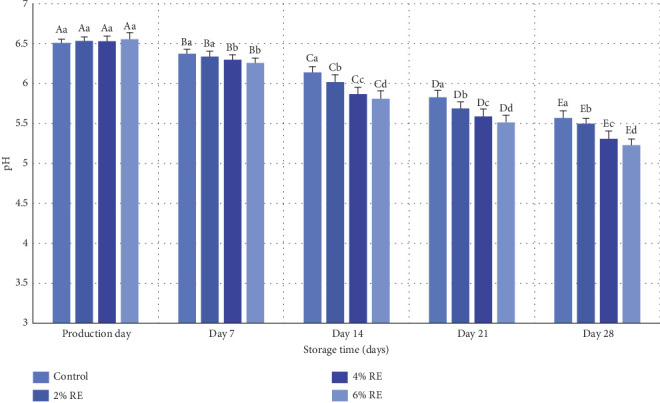
Comparison of the average pH values of fermented milk treatments during 28 days of storage time in the refrigerator. Different small letters denote significant differences among treatments at the same intervals, while different capital letters highlight significant differences within each treatment during storage.

The variance analysis conducted on the dataset revealed statistically significant effects (*p* ≤ 0.05) of treatment, storage duration, and their interaction with the pH levels of fermented milk. As depicted in Figure 2, the initial pH values of the fermented milk samples ranged from 6.51 to 6.55 on the day of production, with no statistically significant differences among the samples. However, over the 28-day refrigerated storage period, all samples exhibited a significant decline in pH values (*p* ≤ 0.05). This reduction is primarily attributable to the metabolic activity of probiotic bacteria, which ferment lactose into L-lactic acid and produce other organic acids. Additionally, the enzymatic activity of probiotics, including the breakdown of proteins and the generation of amino acid groups, further contributed to acidification.

The inclusion of RE notably influenced the rate and extent of pH reduction, with higher concentrations of RE (4% and 6%) associated with a greater decline in pH compared to the control sample. By the end of the storage period, the pH values recorded were 5.57 for the control, and 5.50, 5.31, and 5.23 for samples containing 2%, 4%, and 6% RE, respectively. These findings suggest that RE enhanced the metabolic activity and viability of probiotic bacteria, as reflected in the increased acid production.

These results align with prior research on fermented dairy products. Hong et al. [32] and Yeo et al. [33] observed similar trends of pH reduction during storage due to the activity of LAB in fermented milk products. Both studies highlighted the correlation between enhanced bacterial metabolism and decreased pH values [32, 33]. Additionally, Ahmad et al. [30] reported a comparable decline in pH in probiotic-enriched dairy products, particularly when supplemented with apple polyphenols. Ahmad et al. attributed this effect to the bioactive compounds in polyphenols, which stimulated probiotic growth and metabolic activity, mirroring the role of RE observed in the current study. The findings also resonate with the broader literature on natural extracts in fermented foods. The ability of RE to enhance probiotic activity parallels the results of studies on other plant-derived additives, such as apple polyphenols and date extracts, which have been shown to promote probiotic growth and acid production in yogurt and fermented milk [28, 30]. The observed increase in acidity with higher RE concentrations underscores the synergistic role of bioactive compounds in maintaining probiotic viability and metabolic functionality during storage.

In summary, the current study demonstrates that the incorporation of RE not only sustains but also enhances the metabolic activity of probiotics in fermented milk, leading to a controlled reduction in pH over storage. These findings contribute to the growing evidence supporting the use of natural extracts as functional ingredients to improve the quality and stability of probiotic-rich dairy products, aligning with the trends observed in contemporary research on functional foods.

### 3.4. Titratable Acidity of Fermented Milk

**Figure 3:**
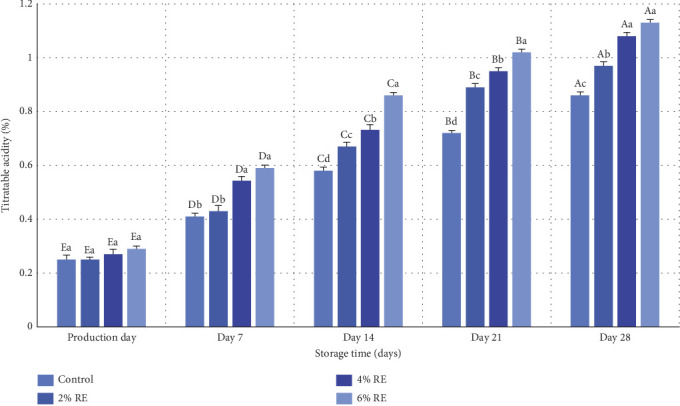
Comparison of average values of titratable acidity (% of L-lactic acid) of fermented milk treatments during 28 days of storage time in the refrigerator. Different small letters denote significant differences among treatments at the same intervals, while different capital letters highlight significant differences within each treatment during storage.

The results of variance analysis revealed statistically significant effects (*p* ≤ 0.05) of treatment, storage duration, and their interaction on the titratable acidity of fermented milk.  Figure 3 presents the changes in mean titratable acidity values for various probiotic-bacteria-fermented milk samples over a 28-day refrigerated storage period. On the day of production, no significant differences in acidity levels were observed among the samples, with values ranging between 0.24% and 0.29% (expressed as L-lactic acidcontent). However, as storage progressed, a significant increase in titratable acidity (*p* ≤ 0.05) was evident in all samples. This trend was most pronounced in samples containing higher concentrations of RE, with the 6% and 4% RE-enriched samples exhibiting the highest acidity levels on day 28 (1.13% and 1.08%, respectively), compared to the control sample, which had the lowest acidity (0.86%).

The observed increase in acidity over the storage period corresponds to the metabolic activity of probiotic bacteria, including their proliferation, fermentation of lactose, and production of metabolites such as L-lactic acid. This aligns with the inverse relationship between pH and titratable acidity observed in the samples. Notably, the addition of RE enhanced the viability and metabolic functionality of probiotics, as evidenced by the accelerated fermentation of sugars and subsequent acid production in samples enriched with higher RE concentrations. These findings are consistent with prior research. Jang et al. [34] reported similar patterns in probiotic yogurt samples, where pH decreased and titratable acidity increased significantly during storage. Their study also demonstrated that the inclusion of ginseng extract heightened the rate of acidification compared to control samples without the extract [34]. This parallels the role of RE in the current study, where the bioactive compounds in the extract likely contributed to enhanced bacterial metabolism and acid production.

The results of this study further substantiate the potential of plant-derived bioactive compounds, such as those found in RE, to improve the functional properties of probiotic dairy products. By promoting probiotic viability and metabolic activity, these natural additives can enhance the acidification process and contribute to the overall stability and quality of fermented milk products during storage.

### 3.5. The Amount of Acetic Acid in Fermented Milk

**Figure 4:**
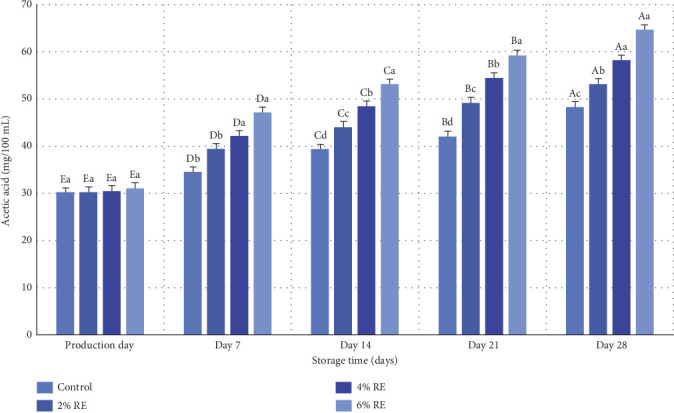
Comparison of average amounts of acetic acid (mg/100 mL) of fermented milk treatments during 28 days of storage in the refrigerator. Different small letters denote significant differences among treatments at the same intervals, while different capital letters highlight significant differences within each treatment during storage.

The results of variance analysis revealed statistically significant effects (*p* ≤ 0.05) of treatment, storage duration, and their interaction on the acetic acid content of fermented milk. As shown in Figure 4, while there was a slight increase in acetic acid levels on the production day attributable to the introduction of varying concentrations of RE, no statistically significant differences were observed among the samples at this stage. However, during the 28-day refrigerated storage period, a significant increase in acetic acid content was recorded in all samples (*p* ≤ 0.05), driven by the metabolic activity of probiotic bacteria. Notably, the control sample exhibited the lowest rate of acetic acid production, whereas the sample containing 6% RE demonstrated the highest rate. Specifically, acetic acid levels rose from an initial 16.30 and 31.03 mg/100 mL in the control and 6% RE samples, respectively, to 27.48 and 64.64 mg/100 mL by the end of the storage period.

The increase in acetic acid levels during storage serves as a marker of the fermentation process, directly reflecting the metabolism of carbon sources and sugars in the system. Acetic acid, a critical metabolite produced by probiotic bacteria, contributes to the antimicrobial properties of fermented dairy products. This observation aligns with the findings of Šušković et al. [35], who highlighted the role of acetic acid in enhancing the functional properties of probiotics. Similar trends in acetic acid production have been reported in related studies. Hassan Mousa et al. [36] documented acetic and lactic acid production in milk fermented with *Saccharomyces boulardii* and lactobacilli, enriched with kiwi juice, underscoring the dependence of acetic acid levels on probiotic activity. Their findings corroborate the current study's results, demonstrating that acetic acid accumulation is closely linked to the metabolic processes of probiotics [36]. Further supporting evidence is provided by Hassan Mousa et al. [37], who observed elevated acetic acid production in fermented milk containing wheat seed juice compared to control samples. Similarly, Massoud et al. [27] reported a direct association between probiotic bacterial activity and increased acetic acid content in probiotic yogurt.

Collectively, these findings underscore the critical role of probiotic bacteria in acetic acid production during fermentation. The enhanced acetic acid levels observed in the current study, particularly in samples enriched with higher concentrations of RE, highlight the potential of natural extracts to amplify probiotic metabolic activity, thereby improving the functional and antimicrobial properties of fermented milk products.

### 3.6. Measurement of L-Lactic Acid in Fermented Milk

**Figure 5:**
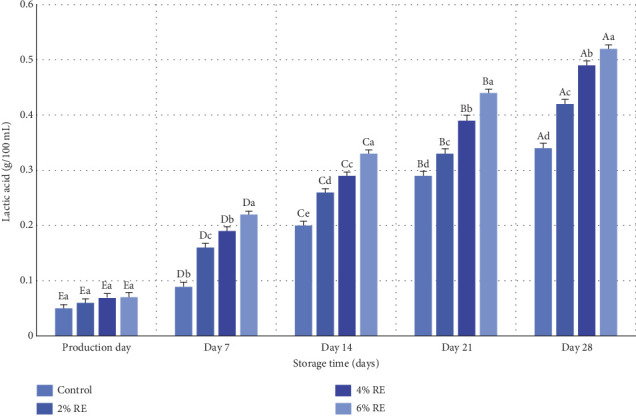
Comparison of the average values of L-lactic acid (g/100 mL) of fermented milk treatments during 28 days of storage time in the refrigerator. Different small letters denote significant differences among treatments at the same intervals, while different capital letters highlight significant differences within each treatment during storage.

The variance analysis results revealed statistically significant effects (*p* ≤ 0.05) of treatment, storage duration, and their interaction with the L-lactic acid content of fermented milk.  Figure 5 illustrates the mean variations in L-lactic acid levels across the probiotic-bacteria-fermented milk samples. On the production day, L-lactic acid levels ranged from 0.05 to 0.07 g/100 mL, with no significant differences among the samples. However, as storage progressed, a marked increase in L-lactic acid content was observed across all samples, attributable to the metabolic activity of LAB and lactose fermentation. This effect was particularly pronounced in samples enriched with RE, especially at 6% and 4% concentrations, which exhibited significantly higher L-lactic acid production compared to the control group (*p* ≤ 0.05). By the 28th day of storage, the control sample demonstrated the lowest L-lactic acid content (0.34 g/100 mL), whereas the 6% and 4% RE samples exhibited the highest levels (0.52 and 0.49 g/100 mL, respectively).

The observed increase in L-lactic acid during storage aligns with prior studies examining the role of LAB in fermented dairy products. For example, Massoud et al. [27] documented a similar rise in lactic acid content during the 21-day storage of probiotic yogurt, underscoring the sustained metabolic activity of probiotics over time. Lee et al. [38] further demonstrated that incorporating bioactive compounds from *Cudrania tricuspidata* into fermented milk enhanced the production of lactic and malic acids, particularly at higher concentrations. This observation mirrors the current study's findings, where increased RE concentrations facilitated more outstanding lactic acid production [38]. Additionally, Hassan Mousa et al. [37] reported a notable increase in lactic acid levels in yogurt enriched with wheat seed water, highlighting the potential of natural additives to bolster probiotic metabolism and organic acid synthesis. These findings resonate with the trends observed in the present study, where the inclusion of RE enhanced probiotic viability and functionality, leading to augmented lactic acid production.

Taken together, these results emphasize the pivotal role of LAB in organic acid production during the storage of fermented milk products. The enhancement of L-lactic acid content by RE supplementation further underscores the value of bioactive plant-based additives in optimizing the functional and biochemical properties of probiotic-enriched dairy products.

### 3.7. Fermented Milk Protein Content

**Figure 6:**
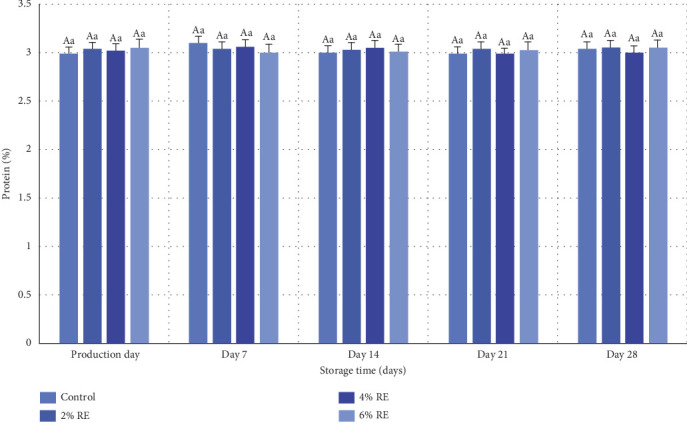
Comparison of average protein values (%) of fermented milk treatments during 28 days of storage time in the refrigerator. Different small letters denote significant differences among treatments at the same intervals, while different capital letters highlight significant differences within each treatment during storage.

The variance analysis results revealed that neither treatment, storage duration, nor the interaction between treatment and storage duration had a statistically significant effect on the protein content of fermented milk (*p* ≥ 0.05). As illustrated in Figure 6, the inclusion of varying concentrations of RE did not lead to any notable alterations in the protein content of goat milk fermented with a mixture of probiotic bacteria. Additionally, no significant changes in protein content were observed over the 28-day refrigerated storage period.

These findings align with previous studies that similarly reported no significant impact of natural extracts on protein content in probiotic dairy products. For instance, Almosawi and Hassan [39] observed that the incorporation of date extract at varying concentrations did not significantly influence the protein content of probiotic fermented milk. Likewise, Abdollahzadeh et al. [28] and Atwaa et al. [16] reported analogous results in their investigations of date extract and fennel extract, respectively, in probiotic yogurt formulations. Their studies demonstrated that these additives, despite their bioactive properties, did not alter the protein composition of the fermented milk products [16, 28]. In agreement with these observations, Jang et al. [34] found no statistically significant differences in protein content between probiotic yogurt samples supplemented with ginseng extract and control samples. This consistency across various studies reinforces the conclusion that the addition of bioactive extracts, including RE, does not significantly impact the protein content of probiotic-fermented dairy products [34].

Overall, the results suggest that the protein content of fermented milk remains stable regardless of the incorporation of plant-based extracts or variations in storage conditions. This stability underscores the robustness of protein composition in probiotic dairy formulations and highlights that functional enhancements by natural additives are more likely to influence other compositional or biofunctional attributes rather than protein content.

### 3.8. The Content of Reducing Sugars in Fermented Milk

**Figure 7:**
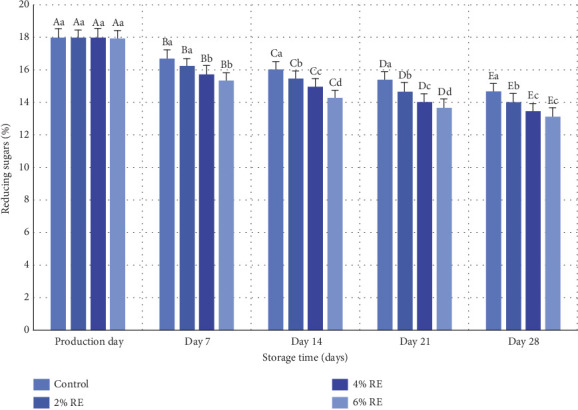
Comparison of the average amount of reducing sugars (%) of fermented milk treatments during 28 days of storage time in the refrigerator. Different small letters denote significant differences among treatments at the same intervals, while different capital letters highlight significant differences within each treatment during storage.

The variance analysis revealed a statistically significant effect (*p* ≤ 0.05) of treatment, storage time, and the interaction between treatment and storage time on the content of reducing sugars in fermented milk.  Figure 7 illustrates the average variations in reducing sugar levels across different probiotic fermented milk samples. On the production day, the reduced sugar content ranged from 17.90% to 17.97% among the samples, with no substantial differences observed. However, during the 28-day refrigerated storage period, a significant decline in reducing sugars was noted in all treatment groups (*p* ≤ 0.05). The most pronounced reduction was observed in samples enriched with 6% and 4% RE, which exhibited final reducing sugar levels of 13.12% and 13.46%, respectively, by the end of the storage period. In contrast, the control sample retained the highest reducing sugar content on the final day (14.66%).

The reduction in reducing sugars during storage can be attributed to their consumption by probiotic bacteria, which metabolize sugars such as glucose and fructose to produce organic acids, particularly lactic acid. This process is central to the lactic acid cycle and facilitates cellular ATP production, which supports the growth and activity of probiotic bacteria. Consequently, as the probiotic population expands, the concentration of reducing sugars diminishes significantly. Samples with higher concentrations of RE exhibited a more pronounced reduction in reducing sugars, indicating that RE may enhance cellular metabolism in probiotics, potentially through its bioactive components. These findings align with prior research, such as the study conducted by Hassan Mousa et al. [37], which documented a significant decrease in reducing sugar levels in fermented milk samples over storage time. Their research also demonstrated that the incorporation of wheat seed water intensified sugar consumption in fermented milk, consistent with the present study's observation of increased sugar utilization in samples containing higher concentrations of RE [37]. Collectively, these results underscore the role of probiotic metabolism in depleting sugar sources during fermentation and storage, while also highlighting the influence of bioactive additives, such as RE, in promoting this metabolic activity.

### 3.9. Evaluation of the Percentage of Dissolved Solids (Brix)

**Figure 8:**
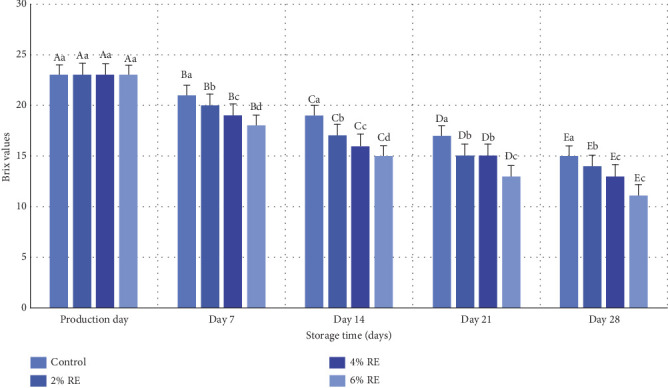
Comparison of the average Brix values of fermented milk treatments during 28 days of storage in the refrigerator. Different small letters denote significant differences among treatments at the same intervals, while different capital letters highlight significant differences within each treatment during storage.

The variance analysis revealed a statistically significant effect (*p* ≤ 0.05) of treatment, storage duration, and the interaction between treatment and storage duration on the dissolved solids content, expressed as Brix degree, in fermented milk. The Brix degree reflects the weight percentage of dissolved solids relative to the total weight of the solution, with higher Brix values, indicating greater solid concentrations and reduced proportions of dissolved water. As depicted in Figure 8, the Brix levels of probiotic fermented milk samples showed no significant differences on the production day, with all samples, including the control, exhibiting a Brix value of 23°. However, during the subsequent 28-day refrigerated storage period, a significant reduction in Brix levels was observed across all samples (*p* ≤ 0.05). This decline is primarily attributed to the metabolic activity of probiotics, which utilize sugars present in the milk and RE for fermentation, converting these sugars into secondary metabolites such as acetic acid and L-lactic acid. Notably, samples enriched with higher levels of RE exhibited more pronounced probiotic activity, leading to greater fermentation intensity and a steeper decline in Brix values. By the end of the storage period, the control sample retained the highest Brix value (15°), whereas the sample containing the highest concentration of RE (6%) exhibited the lowest Brix value (11°).

These findings are consistent with previous studies that have documented similar trends in the reduction of dissolved solids during storage due to probiotic metabolic activity. For example, Yeo et al. [33] and Hong et al. [32] reported significant declines in Brix levels in fermented milk samples over time, a phenomenon linked to the consumption of sugars by LAB during fermentation. Additionally, Lee et al. [38] demonstrated that the initial incorporation of *Cudrania tricuspidata* bioactive compounds in fermented milk samples did not significantly impact the initial Brix levels; however, the metabolic processes during storage led to noticeable reductions in dissolved solids. These studies align with the current findings, reinforcing the role of probiotic metabolism and bioactive compound addition in influencing the composition of fermented milk during storage.

### 3.10. Viscosity Evaluation

**Figure 9:**
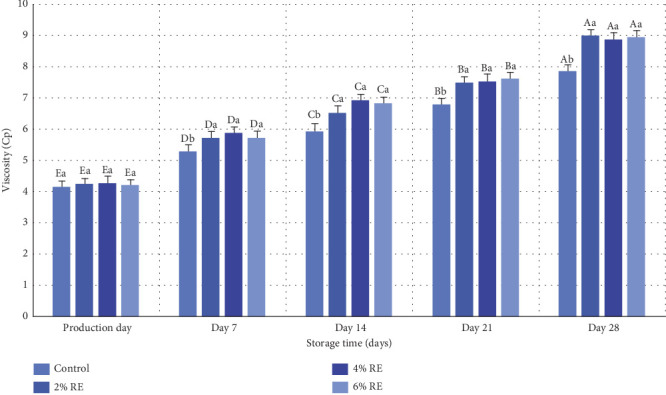
Comparison of average viscosity index of fermented milk treatments during 28 days of storage in the refrigerator. Different small letters denote significant differences among treatments at the same intervals, while different capital letters highlight significant differences within each treatment during storage.

Viscosity is a critical quality attribute of fermented products, influencing consumer perception of product quality [40]. This property is shaped by various factors, including milk heating treatments, ambient temperature, acidity, and the selection of starter cultures. The variance analysis in the present study demonstrated statistically significant effects (*p* ≤ 0.05) of treatment, storage duration, and the interaction between treatment and storage duration on the viscosity of fermented milk. As illustrated in Figure 9, the viscosity of fermented milk samples ranged from 4.15 to 4.27 cP on the production day, with no significant differences between samples. Over the 28-day storage period, a substantial increase in viscosity was observed across all samples (*p* ≤ 0.05), culminating in values between 84.7 and 95.8 cP. Notably, the control sample exhibited the smallest increase, whereas samples enriched with RE, particularly at higher concentrations, experienced more pronounced viscosity enhancements over time.

The increase in viscosity during storage can be attributed to the production of EPSs by probiotic bacteria. These bacteria possess beta-oligosaccharide enzymes that synthesize significant amounts of oligosaccharides, which contribute to viscosity by acting as thickeners and enhancing the textural properties of fermented products [41]. Probiotic bacteria capable of producing EPSs are known to influence rheological properties positively, although the relationship between EPS production and product viscosity is complex and nonlinear [42]. The observed trends align with those reported in previous studies. For example, Almosawi and Hassan [39] noted that the addition of date extract improved the viscosity of fermented milk products, primarily by enhancing the viability of probiotic bacteria, although further increases in extract levels did not yield additional significant changes. Similarly, Matijašić et al. [43] documented a notable rise in viscosity during storage, attributed to accelerated fermentation and the production of secondary metabolites. Hong et al. [32] also reported significant increases in viscosity for probiotic fermented milk products over time, emphasizing the role of storage duration and fermentation dynamics.

In conclusion, the findings of this study demonstrate that the addition of RE and extended storage time significantly enhances the viscosity and metabolic activity of probiotic bacteria in fermented milk, aligning with previous research on the impact of probiotics and bioactive compounds on fermented product characteristics.

### 3.11. Antioxidant Activity of Fermented Milk

Oxidative degradation is a critical process that adversely affects the flavor, bioactive constituents, and nutritional value of food products while generating potentially harmful oxidation byproducts. Mitigating this destructive reaction in food systems necessitates the incorporation of antioxidants, a strategy widely acknowledged for its effectiveness [44]. The DPPH-free radical scavenging assay, commonly employed to evaluate the free radical quenching capacity of samples, is particularly effective for assessing antioxidant activity. Natural antioxidants, abundant in plant extracts, exhibit strong potential to donate hydrogen atoms or electrons to free radicals. This activity is primarily attributed to their high content of bioactive compounds, especially polyphenols, which are recognized for their substantial antioxidant properties [45].

**Figure 10:**
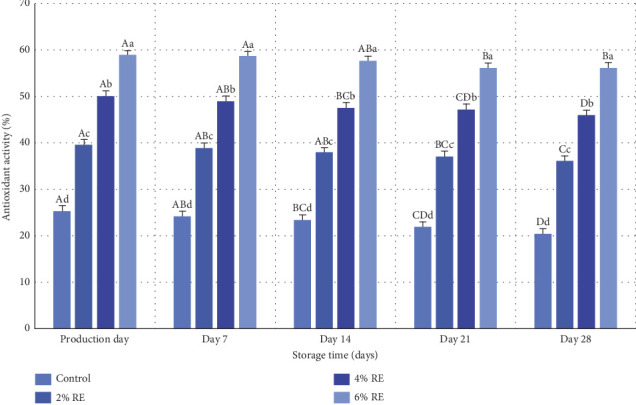
Comparison of the average values of antioxidant activity (%) of fermented milk treatments during 28 days of storage time in the refrigerator. Different small letters denote significant differences among treatments at the same intervals, while different capital letters highlight significant differences within each treatment during storage.

In the present study, the antioxidant activity of fermented milk samples, enriched with varying concentrations of RE, was assessed using the DPPH method, with results presented in Figure 10. Statistical analysis revealed significant effects (*p* ≤ 0.05) of treatment, storage time, and the interaction between these factors on the antioxidant activity of fermented milk. Consistent with prior studies, samples containing higher levels of RE exhibited significantly enhanced antioxidant activity compared to the control on all days of the study (*p* ≤ 0.05). This aligns with findings by Wanyo et al. [46] and Walter and Marchesan [47], who reported that RE's bioactive compounds, endowed with potent antioxidant potential, significantly improve the antioxidant properties of food products upon incorporation.

Additionally, milk components, including catalase enzymes, serum proteins, lactic acid, and probiotic bacteria, contribute to antioxidant activity, facilitating the development of antioxidant-rich fermented products [48]. The incorporation of RE further enhances this activity by improving the viability of probiotic bacteria within the fermented milk matrix. However, a gradual reduction in antioxidant activity during storage, observed in the present study, can be attributed to the oxidation of phenolic compounds in RE and the declining population of probiotic bacteria (*p* ≤ 0.05). These findings are corroborated by Mahdavi Adeli et al. [49], who demonstrated that the addition of Shirazi thyme and oregano extracts significantly increased the antioxidant activity of probiotic yogurt, despite a gradual decline over the storage period. Similarly, Ahmad et al. [30] reported enhanced antioxidant activity in probiotic yogurt samples supplemented with apple polyphenols, followed by a progressive reduction during storage.

The present study also revealed that samples with the highest RE concentration (6%) consistently exhibited superior antioxidant activity throughout the storage period. On the production day, antioxidant activity ranged from 25.33% to 58.94%, decreasing to 20.48%–55.46% by the final day of storage. This trend is consistent with the findings of Abdollahzadeh et al. [28], who observed an enhancement in the antioxidant activity of fermented milk through the incorporation of date extract. Collectively, these results emphasize that RE is a promising additive for improving the antioxidant properties of fermented milk, aligning with broader research trends on the use of plant extracts in functional dairy products.

### 3.12. Evaluation of Color Indicators of Fermented Milk

The results of the variance analysis demonstrated a statistically significant effect (*p* ≤ 0.05) of the applied treatment on the color parameters of fermented milk. The influence of varying RE (rose extract) levels on color attributes, including luminosity (*L*∗), yellow-blue chromaticity (*b*∗), and red-green chromaticity (*a*∗), is presented in Table 2. As shown, increasing RE levels in the samples led to a reduction in luminosity intensity (*L*∗) alongside increases in both red (*a*∗) and yellow (*b*∗) chromaticities. Specifically, the control sample exhibited the highest *L*∗ value (71.86), with the lowest *a*∗ (−2.91) and *b*∗ (31.55) values, whereas the sample with the highest RE concentration (6%) showed the lowest *L*∗ value (67.93), coupled with the highest *a*∗ (1.63) and *b*∗ (37.51) values.

The decrease in luminosity with increasing RE levels can be attributed to the accumulation of pigments inherent in the extract. As the pigment concentration increased, the natural brightness of the fermented milk samples decreased. These findings align with those of Abdollahzadeh et al. [28], who observed reduced luminosity and heightened yellow chromaticity in fermented milk samples enriched with date extract. Similarly, Atwaa et al. [16] reported that the incorporation of fennel extract into probiotic yogurt led to a decrease in luminosity intensity (*L*∗) and an increase in yellow chromaticity (*b*∗). The observed trends suggest that the incorporation of RE at higher concentrations significantly alters the color characteristics of fermented milk, particularly in reducing brightness and enhancing chromaticity. These changes, driven by the pigments in RE, are consistent with prior research demonstrating the impact of natural plant extracts on the color properties of dairy products. Such alterations in color attributes have implications for consumer acceptance and the sensory qualities of the final product.

### 3.13. Sensory Evaluation

**Figure 11:**
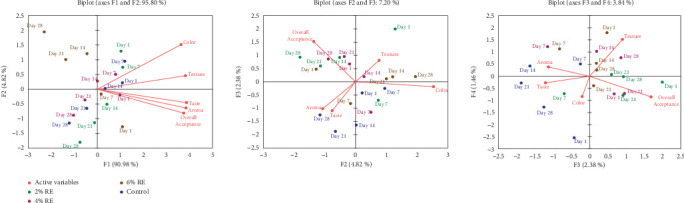
The PCA model for various sensory properties, including color, texture, taste, aroma, and overall acceptance affected by various concentrations of rice extract (RE) of the yogurt, explored in two intervals, based on the Pearson correlation matrix, and considering up to five factors. Significant components were selected based on eigenvalues > 1 (Kaiser's criterion), with variables contributing meaningfully identified by loadings exceeding ± 0.5. A significance level of *α* = 0.05 was applied to ensure the statistical validity of the results.

The findings of the present study, as depicted in Figure 11, reveal how fermentation time and RE concentration influence the sensory properties of fermented goat milk. Using PCA, the relationships between sensory attributes—color, texture, taste, aroma, and overall acceptance—and the progression of sample characteristics over time and RE concentration were explored. The PCA biplots provide insights into these dynamics, linking the results to previous research on RE incorporation into dairy products.

The first principal component (F1) accounted for 90.98% of the variance, indicating its strong correlation with sensory attributes, particularly color and texture, which emerged as the primary drivers of sensory differentiation. Higher RE concentrations (4% and 6%) in samples evaluated on Day 28 aligned positively along the F1 axis, signifying improved scores for these attributes. These findings are consistent with those of Evadewi and Tjahjani [50], who observed that adding black RE to yogurt enhanced its color and texture, contributing to its functionality as an antioxidant-rich product.

Early-stage samples (Day 1 and Day 7) clustered near the origin, suggesting minimal differentiation in sensory attributes at the beginning of fermentation. However, as fermentation progressed, sensory scores improved significantly, with the greatest differentiation observed in Day 28 samples. This trend reflects the synergistic effects of longer fermentation times and higher RE concentrations, similar to the observations of Viswanathan and Shahbazi [51], who reported improved quality and acceptability in yogurt enriched with RE, which also supported probiotic viability.

The second principal component (F2), explaining 7.20% of the variance, highlighted additional patterns, particularly the role of overall acceptance as a secondary driver of sensory differentiation. Samples with 4% and 6% RE concentrations at later fermentation stages aligned positively with F2, demonstrating enhanced overall acceptability. Kiss Firmino Dourado Costa et al. [52] similarly found that fermented RE s inoculated with probiotics achieved high sensory acceptance, particularly for aroma and texture.

The third and fourth components (F3 and F4), though accounting for less variance (3.84%), revealed unique contributions of secondary sensory attributes. Texture and overall acceptance strongly aligned with F4, while taste and aroma displayed more variable effects along F3. These findings suggest that while flavor and aroma are important, visual and tactile attributes such as color and texture play a more dominant role in determining consumer preferences. Montagner and Storck [53] reported similar results, finding that RE ice cream displayed favorable sensory properties, particularly in terms of texture and color.

The study underscores the critical influence of fermentation time and RE concentration on sensory properties. Samples containing higher RE concentrations (4% and 6%) consistently demonstrated improved sensory scores, particularly for color, texture, and overall acceptance. These results align with the work of Mohammadi et al. [54], who demonstrated the potential of rice bran protein as a functional substitute in low-fat desserts, enhancing sensory properties and acceptability. Similarly, Ismail et al. [55] observed that Rayeb milk produced from a 50:50 mixture of cow and rice milk, when supplemented with honey, achieved the highest sensory evaluation scores, emphasizing the positive role of rice ingredients in enhancing flavor and overall acceptability.

The PCA results highlight clear trends in how fermentation time and RE concentration shape sensory properties in fermented goat milk yogurt. Longer fermentation periods (up to 28 days) and higher RE concentrations (4% and 6%) synergistically enhanced sensory attributes, particularly color, texture, and overall acceptance. These findings contribute to a growing body of evidence supporting the incorporation of RE s into dairy products to improve both sensory and functional qualities. The current results align with broader trends in functional food development, demonstrating that RE not only enhances nutritional value but also significantly improves consumer satisfaction, offering promising applications in the dairy and dairy-alternative industries.

## 4. Conclusion

This study evaluated the effects of RE at varying levels on the physicochemical, functional, sensory, and probiotic viability aspects of fermented milk using *Lc. casei* and *Lpb. plantarum*. Over 28 days of refrigerated storage, probiotic counts declined from Day 7, but RE supplementation (2%–6%) significantly improved bacterial survival.

RE addition also increased lactic and acetic acid levels; reduced pH, sugars, and Brix; and raised titratable acidity. These changes, along with enhanced EPS production, led to greater viscosity in enriched samples. Color was also impacted, with RE, especially at higher concentrations, causing reduced brightness and increased redness and yellowness due to its bioactive compounds.

Probiotic viability and RE's bioactives boosted antioxidant activity, showing a strong positive correlation with RE concentration. Sensory analysis found the 2% RE samples most desirable, followed by 4% and the control, while the 6% RE sample scored lowest.

Overall, 4% RE was optimal for improving probiotic viability and functional qualities in fermented milk. Future research should explore RE's effects in different probiotic strains, food systems, and storage conditions, along with mechanism studies, concentration optimization, and assessments of health benefits, scalability, sustainability, and consumer acceptance.

## Figures and Tables

**Table 1:** The results of evaluating the activity of probiotic strains.

**Specification**	***Lc. casei***	***Lpb. plantarum***
Acid resistance	6 log CFU/mL	6 log CFU/mL
Resistance to bile salts	3.3%	3%
Resistance to gastric juice (pepsin and trypsin)	6 log CFU/mL	6 log CFU/mL
Lack of hemolytic activity	Negative	Negative
L-arginine hydrolysis	Negative	Negative
Enumeration of probiotic microorganisms	6 log CFU/mL	6 log CFU/mL

**Table 2:** Variance analysis of colorimetric indices of fermented milk treatments.

**Samples**	***L*** ⁣^∗^	***b*** ⁣^∗^	***a*** ⁣^∗^
Control	71.86 ± 0.62^a^	31.55 ± 0.88^d^	−2.91 ± 0.19^d^
2% extract	70.69 ± 0.58^ab^	34.36 ± 0.79^c^	0.14 ± 0.15^c^
4% extract	69.38 ± 0.91^bc^	35.92 ± 0.65^b^	0.92 ± 0.27^b^
6% extract	67.93 ± 0.73^c^	37.51 ± 0.81^a^	1.63 ± 0.21^a^

*Note:* The presence of at least one common letter suggests no statistically significant difference between the values in each column.

## Data Availability

The data supporting the findings of this study are available from the corresponding author upon reasonable request.
